# In Vivo Measurement of UVA Protection Factor (ISO 24442:2011) Across 38 Different SPF 50 and SPF 50+ Sunscreens Reveals High Heterogeneity and Limitations in the Level of Protection

**DOI:** 10.1111/phpp.70092

**Published:** 2026-04-12

**Authors:** Antony Young, Caroline Tricaud, Françoise Bernerd, Yipin Yuan, Pascale Renoux, Marion Sensenbrenner, Ann‐Laure Demessant, Caroline Le Floc'h, Martin Josso, Philippe Andres

**Affiliations:** ^1^ St John's Institute of Dermatology, King's College London London UK; ^2^ L'Oréal Research & Innovation Chevilly Larue France; ^3^ L'Oréal Research & Innovation Aulnay sous Bois France; ^4^ La Roche‐Posay Laboratoire Dermatologique Levallois‐Perret France; ^5^ Clipeum Pharma Consulting Peymeinade France

**Keywords:** photoprotection, SPF/UVA‐PF ratio, sunscreen, UVA protection

## Abstract

**Background/Purpose:**

The UVB and UVA components of terrestrial solar ultraviolet radiation (UVR) cause acute and chronic damage to skin. These adverse effects can be reduced by sunscreen application. The sun protection factor (SPF) is primarily a measure of UVB protection against erythema and does not quantify protection from UVA, which is the major component of solar UVR. The study aim was to assess UVA protection in relation to labelled SPF and to determine if this met the EU recommendation for a SPF/UVA‐PF ratio of ≤ 3, as recommended by the EU. Unlike SPF assessment, there is no universal consensus for the determination and labelling of UVA‐PF.

**Methods:**

Thirty‐eight sunscreens with SPF ≥ 50 were selected from Europe, Latin America, Oceania, and Asia. UVA‐PF was determined with the ISO 24442:2011 in vivo method, which assesses the ability of a sunscreen to inhibit persistent pigment darkening induced by UVA radiation in a laboratory environment.

**Results:**

There was variation of UVA‐PF ranging from 3.6 to 46.6. Sixty‐eight percent (26/38) of sunscreens complied with EU recommendations. Thus, about one third failed these standards, with SPF/UVA‐PF ratios varying from 3.1 (borderline failure) to 16.7 (major failure). Failure to comply was observed in several products sold in Europe.

**Conclusion:**

The study demonstrates the limitations of many commercial sunscreens to reach EU standards, with significant variations in UVA protection and a lack of relationship between SPF and UVA‐PF values. This highlights the importance of improving labelling standards to ensure consumers receive consistent and adequate UVA protection, especially for those with UVA‐dependent photodermatoses.

**Trial Registration:**

ClinicalTrials.gov: NCT06068010

## 1 Introduction

Sunscreens play a pivotal role in safeguarding skin against the detrimental effects of solar ultraviolet radiation (UVR). The evolution of their formulations has undergone a remarkable journey, progressing from solar UVB (~290–320 nm) protection in the early years to encompassing broader UVA (320–400 nm) protection and, more recently, addressing the potential harm of visible light (400–700 nm) exposure [1, 2, 3, 4, 5, 6, 7, 8]. These advances reflect increased scientific knowledge of the effects of different wavelengths of terrestrial sunlight and continuous innovation in sunscreen technology.

Throughout the mid‐20th century, sunscreens predominantly targeted UVB protection because this spectral region is the main cause of sunburn (erythema), with compounds like para‐aminobenzoic acid (PABA) and benzophenone‐3 (oxybenzone). In 1978, the first standard method for determination and labelling of the Sun Protection Factor (SPF) was issued by the FDA in the USA (“Proposed Monograph”) and came into widespread use as the indicator of prevention of sunburn. However, the limitations of UVB‐focused sunscreens became increasingly apparent as research highlighted the harmful effects of UVA radiation, including photoageing and an increased risk of skin cancer [9, 10]. This prompted the development of broad‐spectrum sunscreens capable of protecting against both UVB and UVA radiations.

The 1980s marked a significant milestone with the introduction of avobenzone, a filter that offered improved protection against UVA radiation. Avobenzone became a key ingredient in broad‐spectrum formulations that provided protection against both UVB and UVA wavelengths [11]. Concurrently, the use of mineral/inorganic UVR filters like zinc oxide and titanium dioxide has gained prominence due to their broad‐spectrum coverage and excellent safety profile [12, 13, 14]. Increased knowledge of adverse effects of UVA has also resulted in the development of other organic UVA filters such as Mexoryl SX (terephthalylidene dicamphor sulphonic acid), Tinosorb S (Bis‐Ethylhexyloxyphenol Methoxyphenyl Triazine), and more recently Mexoryl 400 (Methoxypropylamino Cyclohexenylidene Ethoxyethylcyanoacetate) [15, 16, 17, 18] and Bis‐(Diethylaminohydroxybenzoyl Benzoyl) Piperazine. New protection indices were therefore developed to specifically inform consumers on UVA protection because SPF fails to quantify UVA protection. Despite the availability of reliable methods, there is no worldwide consensus on how to measure and label the level of UVA protection.

The persistent pigment darkening (PPD) method, as outlined in International Organization for Standardization (ISO) 24,442:2011, is considered the gold standard because it is a biological endpoint with remarkable reproducibility. This method is widely favoured because it predominantly assesses a UVA‐specific endpoint [19, 20].

While standardized in vivo methods, particularly those by ISO and Food and Drug Administration (FDA), do evaluate sunscreen performance, there exists a potential for variability in results. Hence, it is imperative to evaluate multiple products using identical methodologies. The objective of our study was to assess the UVA protection factor (UVA‐PF) as outlined in ISO 24442:2011, of a diverse range of international over‐the‐counter facial sunscreens and evaluate if they meet the European Union (EU) standards.

## 2 Materials and Methods

We selected products among the best sellers of different geographical zones (Europe, Latin America, Oceania and Asia), also considering the formulation type and the filtering system to provide a good representation of the global market.

We collected the following characteristics for each product: type of sunscreen (organic or inorganic (mineral)), hydrophilic to lipophilic composition (oil/water (O/W) or water/oil (W/O)), formulation (cream, gel, fluid), labelled SPF 50 or SPF 50 + .

All products underwent testing using the ISO 24442:2011 in vivo method, which is widely acknowledged as the “gold standard” for measuring UVA‐PF. This method evaluates the minimal persistent pigment darkening dose (MPPDD) observed between 2 to 24 h post‐irradiation from a defined UVA emission spectrum (with less than 0.1% of radiation below 320 nm).

The UVA protection factor (UVA‐PF) is calculated as the ratio of MPPDD with protection to MPPDD without protection.

The tests were conducted across six clinical centres in Brazil, France, Poland and Romania, each validated according to L'Oréal's Research & Innovation standards. Quality control measures were meticulously implemented, with each clinical centre monitoring and testing one or two anonymized formulations on volunteers alongside a standard reference sunscreen formulation (S2) with a mean UVA‐PF of 12.7. ISO 24442:2011 requires that the range of UVA‐PF for the reference standard (S2) tested alongside the products studied should be 10.7 to 14.7.

Volunteer groups comprised individuals with phototypes III and IV, with individual typology angles (ITA°) ranging from 20° to 41° in accordance with the ISO methodology ISO 24442:2011. The number of participants per sunscreen is provided in. Table 1 and Table 2.

**TABLE 1:** Characteristics of evaluated sunscreens.

Attribute	*n* (*N* = 38)	Percentage (%)
Sunscreen Type		
Organic	22	57.9
Organic and Inorganic Combination	13	34.2
Inorganic	3	7.9
SPF Value		
SPF 50+	25	65.8
SPF 50 to 100	13	34.2
UVA Protection		
Claimed on Packaging	29	76.3
Numerical Values Provided	7	18.4
Formulation		
Oil/Water	27	71.1
Water/Oil	9	23.7
Purely Oil‐Based	2	5.3
Product Form		
Fluid	23	60.5
Cream	7	18.4
Gel	6	15.8
Gel Cream	2	5.3
Tinted		
Cream	2	5.3
Fluid	5	13.2
Geography		
Latin America	13	34.2
Europe	20	52.6
Asia	2	5.3
Oceania	3	7.9

**TABLE 2:** Comprehensive results of each product with SPF/UVA‐PF ratio.

Product Code	Zone of Purchase	Filtering system	Emulsion type	Texture Type	UVA‐PF (10 subjects)	Labelled SPF	Ratio SPF/UVA
1	Europe	Organic	W/O	Watery gel	3.6 ± 0.3	50+ (60)	16.7
2	Latin America	Organic	W/O	Watery gel	7.8 ± 1.4	50	6.4
3	Oceania	hybrid	W/O	tinted cream	10.1 ± 1.5	50+ (60)	5.9
4	Europe	Organic	W/O	Gel	10.4 ± 1.8	50+ (60)	5.8
7	Latin America	Organic	W/O	Fluid	11.8 ± 1.5	50	4.2
5	Oceania	Organic	W/O	Cream	12.5 ± 2.1	50+ (60)	4.8
6	Europe	hybrid	W/O	gel‐cream	13.8 ± 2.3	50+ (60)	4.3
8	Europe	mineral	Oil phase	Tinted liquid	15.3 ± 2.1	50+ (60)	3.9
9	Europe	hybrid	W/O	Watery gel	16.4 ± 3.9	50+ (60)	3.7
14	Asia	hybrid	O/W	Cream	17.2 ± 2.2	50+ (51)	3.0
19	Asia	mineral	O/W	Cream	17.9 ± 1	50+ (51)	2.8
23	Latin America	Organic	W/O	Fluid	18.0 ± 3.2	50	2.8
12	Latin America	hybrid	O/W	Fluid	19.6 ± 1.3	50+ (60)	3.1
29	Oceania	Organic	W/O	Fluid	19.8 ± 3.2	50	2.5
15	Europe	Organic	W/O	Fluid	20.3 ± 2.7	50+ (60)	3.0
16	Latin America	Organic	W/O	Fluid	20.3 ± 3.7	60	3.0
11	Latin America	hybrid	W/O	Fluid	20.3 ± 2.1	70	3.4
17	Asia	Organic	W/O	gel‐cream	20.6 ± 4.7	50+ (51)	2.5
30	Latin America	mineral	Oil phase	Tinted liquid	20.8 ± 1.5	50	2.4
20	Europe	Organic	W/O	Cream	21.2 ± 1.3	50+ (60)	2.8
21	Europe	hybrid	W/O	Watery gel	21.5 ± 1.5	50+ (60)	2.8
22	Europe	Organic	W/O	Fluid	21.5 ± 0.8	50+ (60)	2.8
24	Europe	hybrid	O/W	Fluid	21.9 ± 1	50+ (60)	2.7
25	Europe	Organic	W/O	Fluid	22.0 ± 1.6	50+ (60)	2.7
26	Europe	Organic	W/O	Fluid	22.1 ± 2.3	50+ (60)	2.7
27	Europe	Organic	W/O	Fluid	22.2 ± 1.4	50+ (60)	2.7
28	Latin America	Organic	W/O	tinted fluid	22.9 ± 1.3	60	2.6
33	Europe	Organic	W/O	Watery gel	23.9 ± 3.6	50	2.1
31	Europe	Organic	O/W	Fluid	25.6 ± 5.6	50+ (60)	2.3
32	Latin America	hybrid	O/W	Fluid	25.6 ± 3.9	60	2.3
10	Latin America	hybrid	O/W	tinted fluid	28.6 ± 3.6	99	3.5
34	Europe	Organic	W/O	tinted fluid	29.3 ± 5.7	50+ (60)	2.0
35	Europe	Organic	W/O	Fluid	29.5 ± 6.4	50+ (60)	2.0
36	Europe	Organic	W/O	Cream	32.6 ± 4.8	50+ (60)	1.8
13	Latin America	hybrid	O/W	Fluid	32.8 ± 6.4	99	3.0
37	Europe	hybrid	W/O	tinted cream	33.4 ± 5.6	50+ (60)	1.8
18	Latin America	hybrid	O/W	Fluid	34.4 ± 7.2	100	2.9
38	Europe	Organic	W/O	Fluid	46.6 ± 9.1	50+ (60)	1.3

*Note:* SPF/UVA‐PF ratio: values in parentheses indicate the SPF value used for ratio calculation. For products sold in Europe labelled SPF 50+, SPF 60 was applied in accordance with the EU Recommendation of 26 September 2006 (2006/647/EC), which specifies SPF 60 as the minimum value for the SPF 50+ labelling category. For SPF 50+ products marketed in Asia, SPF 51 was applied as the minimum value above the 50+ threshold. Products with exact labelled SPF values (e.g., 60, 70, 99, 100) used their labelled value directly.

EU guidelines recommend that sunscreens maintain a UVA‐PF to SPF ratio of at least 1:3 (i.e., SPF/UVA‐PF ≤ 3) [10]. Therefore, we calculated this ratio for each product. For products sold in Europe, the EU Recommendation of 26 September 2006 (2006/647/EC) specifies that the labelled SPF corresponds to the lowest value of the relevant category range; for products labelled SPF 50+, this minimum value is SPF 60, which was therefore used in the ratio calculation. For products marketed in Asia, a value of SPF 51 was applied as the minimum value above the 50+ threshold applicable in those markets.

The studies adhered to the principles of the Declaration of Helsinki and were recorded in Clinicaltrial.gov website (NCT06068010).

This type of study does not require ethical approval, but each participant provided written informed consent.

## 3 Results

Most products contained only organic sunscreens (22/38, 58%), while others featured a combination of organic and inorganic filters (13/38, 34%), with only three being solely inorganic (3/38, 8%). Notably, the labelled SPF values varied, with 25 products indicating SPF 50+, and 13 specifying exact SPF values ranging from 50 to 100. UVA protection claims were made on the packaging of 29 products, though only seven provided numerical values for this protection, ranging from 28 to 56; the others provided semi‐quantitative/less precise indications.

In terms of formulation, the majority (27/38, 71%) were oil/water formulations, while nine were water/oil formulations and two were purely oil‐based. Among them, twenty‐three were in fluid form, seven were creams, six were gels, and two were gel creams. Additionally, seven products were tinted: two were creams and five were fluids. The products were predominantly marketed in Europe [20], Latin America [13], Asia [2] and Australia [3].

Table 2 presents comprehensive findings. UVA‐PF values ranged from 3.6 to 46.6. Among the products assessed, fourteen registered a UVA‐PF below 20, nineteen fell within the 20 to 30 range, and five exceeded 30. The mean UVA‐PF for all products was 21.4. Across regions, those from Europe exhibited a mean UVA‐PF of 22.6 (range: 3.6 to 46.6), those from Latin America recorded 21.9 (range: 7.8 to 34.4), and those from Asia/Australia reported 16.3 (range: 10.1 to 20.6).

Furthermore, analysis by subgroups of sunscreens showed limited variations between subgroups. Mean UVA‐PF values varied by product type, with fluids/lotions scoring a mean of 24 (range: 11.8 to 46.6), creams 20.7 (range: 10.1 to 33.4), and gels 14.7 (range: 3.6 to 23.9). When considering filter types, products containing solely organic filters had a mean UVA‐PF of 21.1 (range: 3.6 to 46.6), those with only mineral filters 18 (range: 15.3 to 20.8), and combinations of organic and mineral filters mean UVA‐PF of 22.7 (range: 10.1 to 34.4). Additionally, products claiming UVA‐PF protection had a mean UVA‐PF of 21.9 (range: 3.6 to 46.6), while those without such claims had a mean UVA‐PF of 19.9 (range: 10.1 to 33.4).

Of note, 68% (26/38) of sunscreens complied with the SPF/UVA‐PF ratio of ≤ 3 recommended by the EU guidelines for UVA protection. Out of the twelve products that failed to meet this criterion, eight claimed to provide UVA protection. All formulation types were involved: three were watery gel, 1 tinted cream, 1 cream, 1 gel cream, 2 tinted fluids, 1 gel, 3 fluids.

## 4 Discussion

Overall, the PPD method specified in ISO 24442:2011 [18, 19] is widely regarded as the gold standard for evaluating UVA‐PF in sunscreen products due to its biological relevance, consistency, reproducibility, and clinical validation.

It reflects the biological effect of UVA exposure leading to persistent darkening of exposed human skin [20]. Moreover, it has been clinically validated through studies correlating PPD values with actual protection against many types of UVA‐induced skin damage, such as DNA lesion formation, photoimmunosuppression, polymorphous light eruption, and photoageing [21]. It is also sensitive to low levels of protection. Unlike in vitro methods that rely on physical measurements, the in vivo method directly measures the biological response of human skin to UVA radiation, providing a more accurate representation of sunscreen efficacy. It reflects the actual effects of UVA exposure on human skin, taking into account factors such as skin type, melanin content, and environmental conditions, which may not be fully captured by in vitro methods [18, 19].

The EU guidelines [10] stating that sunscreens should maintain a UVA‐PF to SPF ratio of at least 1:3, have also been embraced in Australia and Mercosur nations. Additionally, these regions mandate a critical wavelength (CW) of no less than 370 nm, ensuring that 90% of the formulation's absorption spectrum falls within this range. As a result, dermatologists now advocate sunscreens offering high UVA protection across all skin types [22].

Around only two‐thirds of the tested sunscreens complied with EU guidelines for UVA protection, as indicated by an SPF/UVA‐PF ratio of ≤ 3. There was significant variability in the level of UVA protection, with SPF/UVA‐PF ratios ranging from 1.3 to 16.7 and UVA‐PF values ranging from 3.6 to 46.6.

The failure to comply with EU guidelines was observed in all types of formulations that were tested.

Some studies in Latin America also highlighted the heterogeneity of sunscreen UVA‐PF protection. One in vitro study [23] that examined 34 popular over‐the‐counter sunscreens for both UVA and UVB protection revealed varying levels of UVA protection. Another study [24] focused on major tinted sunscreens on the Brazilian market, showing significant variability in UVA transmittance despite excellent UVB performance. Similarly, a study evaluating broad‐spectrum sunscreens in the US market found that approximately 40% of the products had recommended/required UVA protection [24, 25].

An in vitro CW (90% absorption within 370 nm) study was conducted on 20 sunscreen products commercially available in the United States [24]. It revealed that most met the FDA UVA protection criteria for being labelled as broad‐spectrum, yet almost half (9/20, 45%) failed to meet EU standards [25, 26]. This was also the conclusion of a round‐robin study [27] that assessed UVA‐PF of 4 sunscreens. Thus, the EU standard is a more stringent guarantee of UVA protection than the CW approach, which represents an arbitrary measure of UVA filtration bandwidth rather than a given level of biological protection [16, 27, 28, 29, 30, 31].

## 5 Limitations

Different clinical centres were used to test various formulations, though each adhered to strict quality control processes. Moreover, the ISO method is based on measuring an acute effect (persistent pigment darkening). It is generally acknowledged that there are uncertainties regarding the extent to which this acute effect correlates with protection against long‐term effects, like premature skin ageing and skin cancer.

## 6 Conclusion

Our study highlights the limitations of many commercial sunscreens to reach EU standards, with significant variations in UVA protection and non‐compliant SPF/UVA‐PF ratios. Based on our study results, fluids and creams with organic or mixed filters give the best UVA protection. This underscores the importance of improving UVA efficacy standards to ensure consumers receive consistent and adequate UVA protection, especially for those with UVA‐dependent photodermatoses. Finally, the availability of new UVA filters, new combinations of UVA filters and higher permissible levels of UVA filters to produce sunscreens would help ensure better UVA protection in line with the EU standard.

## Funding

This work is funding by La Roche‐Posay Laboratoire Dermatologique, France.

## Ethics Statement

The authors have nothing to report.

## Consent

Each participant provided written informed consent prior to enrolment.

## Conflicts of Interest

A.‐L. Demessant, C. Le Floc'h are employees of La Roche‐Posay, Laboratoire Dermatologique. C. Tricaud, F. Bernerd, Y. Yuan, P. Renoux, M. Sensenbrenner, and M. Josso are employees of L'Oréal Research & Innovation. A. R. Young and P. Andres received a payment from La Roche‐Posay, Laboratoire Dermatologique for their contributions.

## Data Availability

The data that support the findings of this study are available from the corresponding author upon reasonable request.
